# The Association between High-Caffeine Drink Consumption and Anxiety in Korean Adolescents

**DOI:** 10.3390/nu16060794

**Published:** 2024-03-11

**Authors:** Ji Ann Cho, Soyeon Kim, Haein Shin, Hyunkyu Kim, Eun-Cheol Park

**Affiliations:** 1 Premedical Courses, Yonsei University College of Medicine, Seoul 03722, Republic of Korea; jiann.cho22@med.yuhs.ac (J.A.C.); soyeon.kim22@med.yuhs.ac (S.K.); haein.shin22@med.yuhs.ac (H.S.); 2 Department of Preventive Medicine, Yonsei University College of Medicine, Seoul 03722, Republic of Korea; 3 Institute of Health Services Research, Yonsei University, Seoul 03722, Republic of Korea; 4 Department of Psychiatry, Yonsei University College of Medicine, Seoul 03722, Republic of Korea

**Keywords:** anxiety, caffeine, general anxiety disorder, KYRBS

## Abstract

Background: As excessive caffeine intake may be associated with anxiety disorders, one of the most prevalent mental illnesses among adolescents globally, this study investigated the association between high caffeine consumption and anxiety in a nationally representative sample of South Korean adolescents. Methods: 46,873 participants from the Korea Youth Risk Behavior Web-based Survey (KYRBS) 2022 were included. The Generalized Anxiety Disorder-7 (GAD-7) questionnaire was used to evaluate anxiety symptoms. Survey questions determined the number of times each participant consumed high-caffeine drinks per week. The chi-square test was used to investigate and compare the general characteristics of the study population, and a modified Poisson regression was used to analyze the relationship. Results: Both male and female participants reporting excessive high-caffeine drink consumption exhibited higher anxiety levels (adjusted prevalence ratio [aPR]: 1.19, 95% confidence interval [CI]: 1.08–1.31 in males; aPR: 1.14, CI: 1.05–1.23 in females). This association remained statistically significant in subgroup analyses, particularly among high school students and those with a shorter sleep duration. The relationship between high-caffeine drink consumption and anxiety strengthened with increasing anxiety levels. Additionally, there was a dose-dependent relationship between the prevalence of anxiety and high-caffeine drinks. Conclusion: High caffeine consumption increases anxiety in South Korean adolescents. This association proved consistent regardless of sex or other socioeconomic factors.

## 1. Introduction

Anxiety disorders are becoming increasingly prevalent mental health conditions worldwide [1]. In the United States, the lifetime prevalence of anxiety disorders among adolescents is now 26.1% among males and 38.0% among females [2]. Generalized anxiety disorder (GAD), a subtype of anxiety disorder, is characterized by uncontrollable, excessive worry and anxiety symptoms that persist for more than six months. GAD symptoms are often ambiguous as compared to those relating to other mental health issues. Furthermore, they may not always be readily apparent owing to coexisting conditions. Existing research indicates that only 14% of adolescents diagnosed with GAD receive a singular diagnosis without any comorbid conditions [3]. Adolescents may find it challenging to distinguish between normal pubertal anxiety and pathological anxiety, especially when experiencing GAD. In particular, adolescents with GAD express greater concerns about their performance compared to those with other anxiety disorders [3]. Consequently, adolescents should be closely monitored, as their anxiety may be overlooked if attributed merely to meticulous behavior during puberty.

Factors that influence GAD in adolescents include depressive symptoms [4], attachment relationships with parents [5], and sleep patterns [6]. The relationship between COVID-19 and GAD has also been gaining increasing attention of late owing to the prominence of mental health issues associated with the recent pandemic. Considering the increasing amount of caffeine that is being consumed by adolescents through coffee and energy drinks [7], the effects that these high-caffeine drinks have on GAD must also be carefully analyzed.

Adolescents constitute a predominant proportion of consumers in the market for caffeinated beverages. In the USA, energy drinks are targeted at teenagers and young adults aged from 18 to 34 [8]. In South Korea, the prevalence of high-caffeine drink consumption among teenagers has significantly increased over recent years. The proportion of students consuming high-caffeine beverages three or more times per week has alarmingly increased from 3.3% in 2015 to 8.0% in 2017, and further to 12.2% in 2019 [9,10,11]. The criteria for high-caffeine drinks are based on the regulations provided by the Korean Ministry of Food and Drug Safety, categorizing beverages containing more than 0.15 mg/mL of caffeine as high-caffeine drinks [12]. The average amount of caffeine included in high-caffeine drinks available in the Korean market was 58.1 mg [13]. The escalating pattern of consuming high-caffeine drinks among Korean teenagers underscores the growing penetration of caffeine-rich products among the youth, prompting a critical need for increased awareness and, potentially, regulatory measures to mitigate the health impacts associated with excessive caffeine intake in adolescents. Although caffeine intake from certain food sources, like coffee, tea, and a few fruits, is considered safe for adolescents, as these foods also contains other compounds that are beneficial to health, like antioxidants [14,15], high-caffeine drinks may induce excessive caffeine intake. Excessive caffeine intake stimulates the nervous system, thereby reducing fatigue and temporarily enhancing concentration and physical capabilities. Hence, it is frequently used to alleviate fatigue and improve focus [16]. However, excessive caffeine intake can also lead to insomnia, headaches, and high blood pressure. Furthermore, it can induce side effects that include an increased heart rate, restlessness, and anxiety. For healthy adults, a daily caffeine intake of up to 400 mg, equivalent to 6 mg/kg body weight for a person weighing 65 kg, does not significantly elevate the risk of such diseases. While no specific recommendations have been proposed for the adolescent population, pre-adolescents are advised to limit their caffeine intake to a maximum of 2.5 mg/kg body weight/day [17]. Because the recommended maximum level for adolescents is significantly lower than for adults, it is difficult to observe this recommendation. Considering that adolescence is a critical period for the development of dietary habits, particular attention should be paid to caffeine consumption in this age group.

The psychological vulnerability of Korean adolescents highlights the need for further research on anxiety symptoms that are manifesting in Korea. Data from the “Korean Children and Adolescents Happiness Index Survey” has indicated a clear decline in students’ reported life satisfaction since 2017. The data exhibits a trend in which students’ reported life satisfaction notably decreases throughout the transition from elementary to high school [18]. Despite Korean youths exhibiting a higher level of life satisfaction than Korean adults, Korean youths also rank lowest in terms of reported life satisfaction among youths in Organization for Economic Cooperation and Development (OECD) countries, recording an average score of 6.6/10 on the Ministry of Health and Welfare’s 2018 Child Status Survey [19]. This value is significantly below the OECD youth average of 7.6. The youth index also reflects the fact that South Korean youths generally experience a good level of overall wellbeing but exhibit higher-than-average stress levels. Ranking 22nd in the world in terms of health, 65% of the Korean youths surveyed reported experiencing excessive amounts of stress, with South Korea recording a suicide rate of 22 per 100,000 youths, a rate which is above the youth index average for self-harm fatalities of 16 per 100,000 [20]. According to the Korea Youth Risk Behavior Web-based Survey (KYRBS) 2020, over half of the adolescents surveyed cited the burden of performance and academic stress as being the single largest cause of stress (*p* = 51.2%, standard error [SE] = 0.6 in males, *p* = 58.4%, SE = 0.5 in females). These statistics suggest that Korean adolescents are highly distressed by academic stress [21].

The Generalized Anxiety Disorder Assessment (GAD-7) was first included as a variable in Korea’s nationwide youth health survey, the KYRBS, in 2020. Kim and Shin [22] also researched the association between sociodemographic factors, negative emotions, physical activity, and GAD. Several studies have reported the relationship between smoking and GAD among Korean adolescents [23,24], and its impact on sleep quality has also been explored [25,26]. Im [27] investigated health-related behavioral variables, such as alcohol consumption, drug use, and smartphone usage, as factors that may potentially be related to GAD. Currently, no studies report on the impact of caffeine intake on GAD in Korean adolescents. Therefore, to fill this gap, this study aimed to investigate the relationship between the consumption of high-caffeine drinks and GAD in Korean adolescents.

## 2. Methods

### Study Population and Data

The data analyzed in this study were obtained from the Korea Youth Risk Behavior Web-based Survey (KYRBS) 2022, which was conducted by the Korean Ministry of Education, the Ministry of Health and Welfare, and the Korean Disease Control and Prevention Agency. This anonymous survey targets middle and high school students, aiming to assess the health status and behaviors of South Korean adolescents, thereby providing foundational data for health policy formulation. The survey is conducted annually in approximately 400 middle schools and 400 high schools. The survey items themselves vary slightly each year, and weight values have been suggested for combined analysis over several years. A total of 46,873 participants, comprising 24,104 males and 22,283 females, engaged in the online survey.

## 3. Measures

### 3.1. GAD-7

The GAD-7 is a self-report questionnaire designed for screening anxiety disorders. It includes seven items that reflect the *Diagnostic and Statistical Manual of Mental Disorders, Fourth Edition* (DSM-IV) symptom criteria for generalized anxiety disorders. The scale has been established as a reliable, valid screening tool for GAD and other anxiety disorders. Each item is rated with a score between 0 and 3, corresponding to a maximum total score of 21. The level of reported anxiety can be divided into four different levels according to the total score: normal (0–4), mild (5–9), moderate (10–14), and severe (15–21). The Korean version of the GAD-7, translated by Seo et al. [28], demonstrated a Cronbach’s α of 0.915. This was combined with a sensitivity of 78.1%, a specificity of 74.6%, a positive predictive value (PPV) of 46.3%, and a negative predictive value (NPV) of 92.4%, with a cutoff score of five. Therefore, participants with a GAD-7 level of five or higher were defined as experiencing anxiety in this study.

### 3.2. High-Caffeine Drink Consumption

To evaluate the study participants’ level of high-caffeine drink consumption, participants answered a question about the frequency of their high-caffeine drink consumption over the past seven days. High-caffeine drink consumption was measured according to the question, “Over the past seven days, how often did you consume high-caffeine drink?” Energy drinks (e.g., Red Bull, Monster Energy), coffee, and coffee-infused beverages (e.g., coffee-flavored milk) were cited as examples of high-caffeine drinks. Five choices were presented as possible answers to this question: 1—none; 2—1~2 times a week; 3—3~4 times a week; 4—5~6 times a week; 5—once every day of the week; 6—twice every day of the week; 7—more than three times a day for a week. Participants were then classified into two groups based on their daily high-caffeine drink intake. Given that the average caffeine content of high-caffeine drinks is 58.1 mg, consuming two servings approximates the recommended daily limit for adolescents, which corresponds to 125 mg/day for a person weighing 50 kg. Therefore, the classification cutoff hinges on whether an individual consumes equal to or more than two servings or more high-caffeine drinks per day. Consequently, participants were categorized into two groups: non-daily to daily caffeine consumers (response options 1–5), identified as the normal consumption group, and multiple times daily caffeine consumers (response options 6 and 7), identified as the excessive consumption group.

### 3.3. Covariates

Age, family structure, and academic grades were included as sociodemographic covariates. Based on family structure, participants were classified as having one parent, both parents, or none. Additionally, health-related covariates, including sweet drink consumption, fast food consumption, alcohol consumption, smoking status, sleep duration, and perceived stress level were considered and adjusted for in the analyses.

### 3.4. Statistical Analysis

Chi-square tests were used to analyze and compare variables. To examine the relationship between high-caffeine drink consumption and anxiety, we conducted a modified Poisson regression analysis after adjusting for covariates. Subgroup analyses were performed to investigate the combined associations between high-caffeine drink consumption and other covariates and anxiety. Participants with anxiety were divided into three groups according to the severity of their anxiety. Differences in the relationship between high-caffeine drink consumption and the three severity groups were subsequently analyzed. The results were presented as prevalence ratios (PR) and 95% confidence intervals (CI) to compare the prevalence of anxiety. The analyses were performed using stratified sampling variables (strata) and weighted variables suggested by the KYRBS. All analyses were carried out using SAS software (version 9.4; SAS Institute, Cary, NC, USA), and *p*-values < 0.05 were considered statistically significant.

## 4. Results

The general characteristics of the study population, stratified by sex, are presented in Table 1. As outlined in the methods section, a total of 46,873 participants comprising 23,677 males and 23,196 females were included in the analysis. Among them, 29.2% of males and 41.6% of females reported experiencing anxiety, as defined by a GAD-7 score ≥5. Both male and female participants who consumed excessive amounts of high-caffeine drinks exhibited a higher prevalence of anxiety.

Table 2 presents the results of the modified Poisson regression analysis investigating the association between high-caffeine drink consumption and anxiety. After adjusting for covariates, participants of both sexes who engaged in excessive consumption of high-caffeine drinks exhibited a higher prevalence of anxiety (adjusted PR: 1.19, 95% CI: 1.08–1.31 in males; adjusted PR: 1.14, CI: 1.05–1.23 in females).

Furthermore, participants with unhealthy habits, such as consuming sweet drinks or fast food, showed higher adjusted PRs than those without such habits. Participants who frequently consumed sweet drinks showed adjusted PRs of 1.07 (95% CI: 1.02–1.12) in males and 1.06 (95% CI: 1.03–1.10) in females. Similarly, participants who frequently consumed fast food showed adjusted PRs of 1.09 (95% CI: 1.04–1.14) in males and 1.09 (95% CI: 1.05–1.12) in females.

After adjusting for covariates, participants who slept six or fewer hours per night showed the highest PR of anxiety compared to those who slept more than eight hours (adjusted PR: 1.29, 95% CI: 1.19–1.40 in males; adjusted PR: 1.20, CI: 1.11–1.30 in females) and those who slept between six and eight hours ranked second (adjusted PR: 1.14, 95% CI: 1.06–1.24 in males; adjusted PR: 1.11, CI: 1.02–1.20 in females).

Additionally, anxiety prevalence within each subgroup tended to increase with participants’ perceived stress levels. To elaborate, among males with a middle and high perceived stress level, adjusted PRs were 3.12 (95% CI: 2.76–3.53) and 8.10 (95% CI: 7.19–9.13), respectively, compared to the low-stress group. This trend persisted within the female group, with adjusted PRs of 3.36 (95% CI: 2.91–3.88) and 8.40 (95% CI: 7.31–9.66), respectively.

The results of the subgroup analyses, performed using the modified Poisson regression model after subgrouping participants based on their characteristics, are presented in Table 3. Adolescents in high school showed statistically significant adjusted prevalent ratios (PRs) across male (adjusted PR: 1.21, 95% CI: 1.08–1.37) and female demographics (adjusted PR: 1.17, 95% CI: 1.07–1.29). The association between high-caffeine drink consumption and anxiety was shown to be statistically significant among adolescents having reported six or fewer hours of sleep per night (adjusted PR: 1.22, 95% CI: 1.10–1.35 in males; adjusted PR: 1.16, 95% CI: 1.07–1.25 in females). The subgroups with a sleep duration of 8 or more hours across both sexes did not show a significant prevalence since the confidence intervals displayed a wide range owing to the small number of participants in each subgroup.

**Figure 1:**
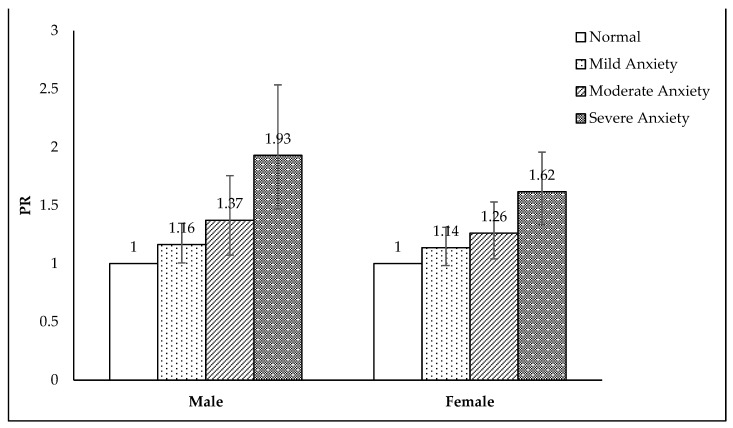
Prevalence ratio (PR) of reported anxiety levels in relation to high-caffeine drink consumption. The reference group refers to individuals who exhibit a normal consumption of high-caffeine drinks.

Figure 1 illustrates the results of the association between high-caffeine drink consumption and various levels of anxiety. The PRs increased as the anxiety level became higher for both male (adjusted PR: 1.16, 95% CI: 1.01–1.35 for mild anxiety; adjusted PR: 1.37, 95% CI: 1.07–1.75, for moderate anxiety; adjusted PR: 1.93, 95% CI: 1.47–2.53 for severe anxiety) and female participants (adjusted PR: 1.14, 95% CI: 0.98–1.31 for mild anxiety; adjusted PR: 1.26, 95% CI: 1.04–1.53 for moderate anxiety; adjusted PR: 1.62, 95% CI: 1.34–1.96 for severe anxiety).

Participants were re-categorized into five detailed subgroups based on their level of high-caffeine drink consumption to analyze the relationship between the doses taken and participants’ existence of anxiety. Table 4 demonstrates the statistically significant differences between adolescents who consumed high-caffeine drinks multiple times per day and those who did not. Adolescents who consumed high-caffeine drinks multiple times per day showed significantly higher adjusted PR among both male (adjusted PR: 1.18, 95% CI: 1.04–1.33 for participants who consumed high-caffeine drinks twice daily; adjusted PR: 1.20, 95% CI: 1.03–1.39 for participants who consumed high-caffeine drinks three or more times a day) and female participants (adjusted PR: 1.16, 95% CI: 1.06–1.28 for participants who consumed high-caffeine drinks twice daily; adjusted PR: 1.32, 95% CI: 0.98–1.26 for participants who consumed high-caffeine drinks three or more times a day).

## 5. Discussion

### 5.1. Association between High-Caffeine Drink Consumption and Anxiety in Korean Adolescents

In this study, GAD scores were found to be associated with the frequency of high-caffeine drink consumption among Korean adolescents. Furthermore, within the high school subgroup, adjusted PRs of anxiety were 1.21 (95% CI: 1.08–1.37) in males and 1.17 (95% CI: 1.07–1.29) in females, indicating the significant impact of high caffeine consumption on the prevalence of anxiety. Additionally, adolescents with a shorter sleep duration displayed a stronger association with anxiety than those who experienced sufficient sleep duration. These results did not differ based on participants’ sex and remained consistent after adjusting for covariates and subgrouping by sociodemographic and health-related variables.

Our findings align with previous studies which demonstrated that symptoms relating to GAD are significantly associated with excessive high-caffeine drink consumption in adolescents [29,30]. As our study indicates that excessive high-caffeine drink consumption is associated to higher GAD scores, we propose that either excessive caffeine consumption leads to anxiety, or adolescents with anxiety exhibit a preference for higher levels of caffeine intake. However, considering that caffeine induces symptoms of anxiety, a condition diagnosed as caffeine-induced anxiety, it is possible to suggest that excessive caffeine consumption induces higher levels of anxiety in Korean adolescents.

### 5.2. Role of Academic Stress and Sleep Duration on Adolescent Anxiety

One remarkable observation from this study is the significant association noted between caffeine consumption and GAD of middle and high school students who reported experiencing reduced sleep duration. Academic stress is the primary cause of stress among Korean adolescents [31]. They often resort to consuming caffeine to sustain an elevated state of arousal and enhance their concentration while studying [32,33]. A survey conducted among Korean adolescents regarding their experience with energy drink consumption reported that 64.6% of respondents consumed these beverages to relieve fatigue, with 69.7% reporting that they consumed these drinks while studying [34]. Therefore, we suggest that Korean adolescents usually consume high-caffeine drinks in an attempt to reduce their academic burden. However, excessive consumption of these drinks also induces anxiety, making these adolescents more susceptible to academic stress. This cycle can create a positive feedback loop between high-caffeine drink consumption and anxiety.

The consumption of such beverages appears to be more prevalent among high school students who are overwhelmed by their academic workload, with middle school students being less affected in this sense. Of notable concern is the fact that the impact of high-caffeine drinks on GAD was found to be significantly higher in both the subgroup of high school students and those who reported experiencing reduced sleep. Adolescents who consume caffeine commonly experience reduced sleep duration and a decline in sleep quality [33,35,36,37]. These results imply that excessive caffeine intake adversely affects sleep, which, in turn, leads to increased GAD scores. The relationship between sleep duration and GAD has already been confirmed in previous studies [25]. These results suggest an increased risk of anxiety among the consumers of such beverages.

Analysis of fractionalized high-caffeine drink consumption revealed that anxiety tended to increase alongside the amount of caffeine consumed. The adjusted PR did not increase uniformly. However, it should also be noted that the recommended caffeine intake for adolescents appears to be weight-dependent, with individual differences observed in terms of the impact of caffeine consumption on different students. These discrepancies can be attributed to the individual effects of caffeine intake on each student.. A notable impact of high-caffeine drinks on anxiety was observed, with a threshold identified at twice-daily consumption, which corresponds to the borderline separating normal and excessive intake levels.

Previous studies have also suggested that the consumption of high-caffeine drinks has a larger impact on adolescents than it does on adults [38]. In addition, caffeine consumption in adolescents can lead to anxiety-related behaviors developing in adults [39]. To reduce the prevalence of anxiety disorders in Korean society as a whole, it is important to be aware of, and to provide education about, the anxiety that can be caused by excessive caffeine consumption.

### 5.3. A Global Perspective on Caffeine Consumption and the Need for Targeted Interventions

In the context of increasing global caffeine consumption, the United States is reported to have the highest total caffeine intake, with more than 80% of its adult population consuming caffeine daily [40]. The average coffee serving size in the United States is approximately 9 ounces. Furthermore, Finland’s per capita coffee consumption, the highest globally, corresponds to an annual average caffeine consumption of approximately 13 kg (28.7 pounds) per person [41]. Given the substantial levels of caffeine consumption documented worldwide, it is imperative to elucidate the correlation between adolescent anxiety and caffeine intake and to establish appropriate health policies for all countries. We contend that the findings of this study apply not only to South Korea but also globally.

Amidst the global growth of the high-caffeine drink market, where Red Bull and Monster Energy hold significant market shares [42], it is presumed that energy drink consumption patterns are largely similar internationally. In 2021, Monster Energy captured a 58% share of South Korea’s energy drink market [43]. Therefore, the association between caffeine intake and anxiety should be considered in the context of similar global consumption patterns.

The specific cause of distress differs across age groups, since middle school students, compared to high school students, were more likely to experience distress from conflicts with parents, peers, or teachers. Conversely, it is shown that high school students were more distressed by academic performance than their middle school counterparts [44]. While the sources of stress vary regarding the age of adolescents, academic burden remains a notable contributor to distress among the entire Korean adolescent population. A study conducted by the Korea Institute for Health and Social Affairs (2015) identified academic factors (32.9%) as the personal anxiety factors that most affect middle and high school students, with frequent changes in educational entrance exams (17.6%) identified as a key factor contributing to societal anxiety [45].

In contrast, in other nations, such as the United States, where youth employment rates are higher, difficult experiences in adolescence are commonly cited as a principal cause for anxiety. A study of New Zealand youth also highlighted a correlation between caffeine consumption and self-assessed stress, anxiety, and depression among secondary school children [29].

### 5.4. Study Limitations and Strengths

This study possesses several limitations. First, cross-sectional data could not establish the causal relationship between high-caffeine drink consumption and the prevalence of anxiety in adolescents. Second, as the database was constructed through a self-reported online survey, there is a possibility of recall bias and participants’ misunderstanding of some questions. Third, as the survey targeted youths specifically, the questionnaire was kept deliberately simple and did not include a specification of the amount of caffeine consumed. This hindered a more precise measurement of participants’ caffeine consumption, although it could have allowed for a more objective assessment. For instance, several types of high-caffeine drinks vary in their caffeine content: each can of Monster Energy contains 100 mg of caffeine per serving, while coffee-flavored milk contains 48 mg. Although both Monster Energy and coffee-flavored milk were presented as sources of caffeine, the caffeine content of the latter does not even correspond to half of the former.

Despite these limitations, this study has several strengths. Although the KYRBS is limited by its cross-sectional database, it encompasses nationally representative sample data, facilitating the extrapolation of results to a broader adolescent population across South Korea. Additionally, the variable of high-caffeine drink consumption, grouped into normal or excessive consumers, was statistically associated with the development of anxiety disorders. Therefore, this metric can be readily and easily applied to assess whether adolescents are consuming excessive caffeine and experiencing anxiety issues.

## 6. Conclusions

In conclusion, this study identified the existence of a relationship between high-caffeine drink consumption and anxiety among Korean adolescents. This association became stronger as anxiety levels and caffeine consumption increased. This association remained consistent with other aspects of the participants. Further research using prospective designs, allowing for a clear assessment of caffeine consumption relative to personal caffeine resistance through objective instruments, should be conducted to further validate the accuracy of these findings.

## Figures and Tables

**Table 1:** Socioeconomic and health-related characteristics of all study participants according to their GAD scores.

Variables		Male (N = 23,677)				Female (N = 23,196)			
		GAD ≥ 5		GAD < 5		GAD ≥ 5		GAD < 5	
		N	(%)	N	(%)	N	(%)	N	(%)
High-Caffeine Drink Consumption									
	Normal	6685	(28.8)	16,499	(71.2)	9365	(41.1)	13,398	(58.9)
	Excessive	235	(47.7)	258	(52.3)	275	(63.5)	158	(36.5)
Age									
	12–15	3556	(27.9)	9187	(72.1)	5275	(41.7)	7364	(58.3)
	16–18	3364	(30.8)	7570	(69.2)	4365	(41.3)	6192	(58.7)
Having Parents									
	Both Parents	4907	(29.4)	11,770	(70.6)	7803	(41.7)	10,896	(58.3)
	Single Parent	301	(30.9)	673	(69.1)	460	(47.1)	516	(52.9)
	None	1712	(28.4)	4314	(71.6)	1377	(39.1)	2144	(60.9)
Sweet Drink Consumption									
	Low	1982	(25.5)	5800	(74.5)	3528	(37.3)	5938	(62.7)
	High	4938	(31.1)	10,957	(68.9)	6112	(44.5)	7618	(55.5)
Fast Food Consumption									
	Low	4692	(27.6)	12,334	(72.4)	6907	(39.6)	10,547	(60.4)
	High	2228	(33.5)	4423	(66.5)	2733	(47.6)	3009	(52.4)
Alcohol Consumption									
	None	3979	(27.3)	10,619	(72.7)	6351	(38.4)	10,189	(61.6)
	Low	2804	(32.1)	5940	(67.9)	3190	(49.3)	3281	(50.7)
	High	137	(40.9)	198	(59.1)	99	(53.5)	86	(46.5)
Smoking Status									
	Never	5975	(28.4)	15,055	(71.6)	8931	(40.6)	13,046	(59.4)
	Ever	945	(35.7)	1702	(64.3)	709	(58.2)	510	(41.8)
Grade									
	High	2723	(28.7)	6780	(71.3)	3544	(39.9)	5337	(60.1)
	Middle	1864	(27.1)	5008	(72.9)	2873	(39.7)	4367	(60.3)
	Low	2333	(32.0)	4969	(68.0)	3223	(45.6)	3852	(54.4)
Sleep Duration									
	>8 h	642	(21.5)	2348	(78.5)	473	(30.0)	1102	(70.0)
	6–8 h	2659	(25.8)	7630	(74.2)	2983	(36.4)	5212	(63.6)
	≤6 h	3619	(34.8)	6779	(65.2)	6184	(46.1)	7242	(53.9)
Perceived Stress Level									
	Low	315	(6.4)	4595	(93.6)	224	(7.4)	2799	(92.6)
	Middle	2085	(20.0)	8363	(80.0)	2358	(25.2)	7004	(74.8)
	High	4520	(54.3)	3799	(45.7)	7058	(65.3)	3753	(34.7)
Total		6920	(29.2)	16,757	(70.8)	9640	(41.6)	13,556	(58.4)

**Table 2:** Adjusted prevalence ratios of anxiety in relation to socioeconomic and health-related covariates among adolescents.

Variables		Male		Female	
		GAD ≥ 5		GAD ≥ 5	
		Adjusted PR	95% CI	Adjusted PR	95% CI
High-Caffeine Drink Consumption					
	Normal	1.00		1.00	
	Excessive	1.19	(1.08–1.31)	1.14	(1.05–1.23)
Age					
	12–15	1.00		1.00	
	16–18	0.99	(0.95–1.03)	0.88	(0.85–0.91)
Having Parents					
	Both Parents	1.00		1.00	
	Single Parent	1.00	(0.90–1.10)	1.00	(0.94–1.07)
	None	0.93	(0.88–0.97)	0.93	(0.89–0.97)
Sweet Drink Consumption					
	Low	1.00		1.00	
	High	1.07	(1.02–1.12)	1.06	(1.03–1.10)
Fast Food Consumption					
	Low	1.00		1.00	
	High	1.09	(1.04–1.14)	1.09	(1.05–1.12)
Alcohol Consumption					
	None	1.00		1.00	
	Low	1.03	(0.98–1.07)	1.13	(1.09–1.17)
	High	1.13	(0.99–1.29)	1.14	(1.00–1.30)
Smoking Status					
	Never	1.00		1.00	
	Ever	1.02	(0.96–1.08)	1.12	(1.06–1.18)
Grade					
	High	1.00		1.00	
	Middle	0.94	(0.90–0.99)	1.01	(0.97–1.04)
	Low	1.02	(0.98–1.07)	1.04	(1.00–1.08)
Sleep Duration					
	>8 h	1.00		1.00	
	6–8 h	1.14	(1.06–1.24)	1.11	(1.02–1.20)
	≤6 h	1.29	(1.19–1.40)	1.20	(1.11–1.30)
Perceived Stress Level					
	Low	1.00		1.00	
	Middle	3.12	(2.76–3.53)	3.36	(2.91–3.88)
	High	8.10	(7.19–9.13)	8.40	(7.31–9.66)

**Table 3:** Subgroup analysis of the association between high-caffeine drink consumption and anxiety stratified by sociodemographic and health-related variables.

Variables		Male			Female		
		High-Caffeine Drink Consumption			High-Caffeine Drink Consumption		
		Normal	Excessive		Normal	Excessive	
		Adjusted PR	Adjusted PR	95% CI	Adjusted PR	Adjusted PR	95% CI
Age							
	12–15	1.00	1.14	(0.97–1.33)	1.00	1.07	(0.95–1.20)
	16–18	1.00	1.21	(1.08–1.37)	1.00	1.17	(1.07–1.29)
Having Parents							
	Both Parents	1.00	1.25	(1.12–1.40)	1.00	1.11	(1.02–1.22)
	Single Parent	1.00	1.05	(0.70–1.59)	1.00	1.38	(1.13–1.68)
	None	1.00	1.08	(0.90–1.31)	1.00	1.14	(0.95–1.37)
Sweet Drink Consumption							
	Low	1.00	1.11	(0.85–1.45)	1.00	1.28	(1.10–1.49)
	High	1.00	1.20	(1.09–1.33)	1.00	1.10	(1.01–1.20)
Fast Food Consumption							
	Low	1.00	1.18	(1.03–1.36)	1.00	1.15	(1.05–1.26)
	High	1.00	1.22	(1.07–1.38)	1.00	1.11	(0.98–1.27)
Alcohol Consumption							
	None	1.00	1.19	(1.02–1.38)	1.00	1.18	(1.06–1.32)
	Low	1.00	1.23	(1.08–1.39)	1.00	1.09	(0.98–1.23)
	High	1.00	0.96	(0.70–1.32)	1.00	1.25	(0.96–1.64)
Smoking Status							
	Never	1.00	1.14	(1.02–1.28)	1.00	1.16	(1.07–1.26)
	Ever	1.00	1.33	(1.13–1.57)	1.00	0.99	(0.83–1.19)
Grade							
	High	1.00	1.25	(1.08–1.43)	1.00	1.01	(0.87–1.16)
	Middle	1.00	1.06	(0.85–1.32)	1.00	1.29	(1.14–1.47)
	Low	1.00	1.23	(1.05–1.44)	1.00	1.13	(1.00–1.28)
Sleep Duration							
	>8 h	1.00	1.06	(0.62–1.83)	1.00	0.81	(0.30–2.18)
	6–8 h	1.00	1.13	(0.88–1.45)	1.00	1.07	(0.82–1.40)
	≤6 h	1.00	1.22	(1.10–1.35)	1.00	1.16	(1.07–1.25)
Perceived Stress Level							
	Low	1.00	2.80	(1.55–5.06)	1.00	1.63	(0.60–4.41)
	Middle	1.00	1.26	(0.92–1.73)	1.00	1.48	(1.07–2.03)
	High	1.00	1.15	(1.04–1.26)	1.00	1.11	(1.03–1.19)

**Table 4:** Results of the modified Poisson regression analysis on participants’ dose-dependency on high-caffeine drink consumption in relation to GAD.

Variables		Male		Female	
		GAD ≥ 5		GAD ≥ 5	
		Adjusted PR	95% CI	Adjusted PR	95% CI
High-Caffeine Drink Consumption					
	None	1.00		1.00	
	<1 time/day	1.00	(0.95–1.04)	1.01	(0.98–1.04)
	once/day	1.03	(0.93–1.14)	1.03	(0.95–1.11)
	twice/day	1.18	(1.04–1.33)	1.16	(1.06–1.28)
	≥3 times/day	1.20	(1.03–1.39)	1.11	(0.98–1.26)

## Data Availability

Data used in this study were from the 2022 KYRBS. Raw data as a whole are available to the public, and the data can be downloaded from the KYRBS official website: https://www.kdca.go.kr/yhs/ (accessed on 28 September 2023).
